# Use of the MLPA Assay in the Molecular Diagnosis of Gene Copy Number Alterations in Human Genetic Diseases

**DOI:** 10.3390/ijms13033245

**Published:** 2012-03-08

**Authors:** Liborio Stuppia, Ivana Antonucci, Giandomenico Palka, Valentina Gatta

**Affiliations:** Department of Oral Sciences, Nano and Biotechnologies, “G. d’Annunzio” University, Via dei Vestini 31, 66013 Chieti, Italy; E-Mails: i.antonucci@unich.it (I.A.); gdpalka@unich.it (G.P.); v.gatta@unich.it (V.G.)

**Keywords:** gene copy number, MLPA, CNV, molecular diagnosis, genetic disease

## Abstract

Multiplex Ligation-dependent Probe Amplification (MLPA) assay is a recently developed technique able to evidence variations in the copy number of several human genes. Due to this ability, MLPA can be used in the molecular diagnosis of several genetic diseases whose pathogenesis is related to the presence of deletions or duplications of specific genes. Moreover, MLPA assay can also be used in the molecular diagnosis of genetic diseases characterized by the presence of abnormal DNA methylation. Due to the large number of genes that can be analyzed by a single technique, MLPA assay represents the gold standard for molecular analysis of all pathologies derived from the presence of gene copy number variation. In this review, the main applications of the MLPA technique for the molecular diagnosis of human diseases are described.

## 1. Background

Although the majority of human hereditary diseases are due to abnormalities in the DNA sequence of specific genes (point mutations), gene deletions or duplications represent a relevant portion (about 5%) of all disease-causing mutations, and in some cases are the most frequent cause of a genetic disease, such as in the cases of Duchenne Muscular Dystropy (DMD) or Spinal Muscular Atrophy (SMA) [1–3]. The correct characterization of gene deletions and duplications is a crucial point in order to identify the genotype phenotype correlation. In fact, entire and partial gene deletions/duplications can produce a completely different phenotypic effect. A complete gene duplication can lead to a disease due to the presence of an extra copy of the gene, while a partial duplication can lead to a loss of function for that gene copy, such as in the case of DMD where duplications affect some exons within the gene, but not the entire gene. Moreover, the complete absence of a protein or the presence of a partially deleted protein, lead in the first case to DMD and in the second one to BMD (see Section 3). In addition, it has been recently demonstrated that the genetic basis of several human diseases is related to the Copy Number Variation (CNV), generally defined as a DNA segment, longer than 1 kb, showing a variable copy number compared with a reference genome [4]. At present, the real proportion of genetic diseases caused by CNVs is unknown, but it may be substantial, when considering that it has been suggested that germline CNVs can also predispose an individual to syndromic malformations [5]. Neither conventional cytogenetic analysis or DNA sequencing is able to detect gene deletions/duplications and CNVs. As a consequence, these mutations must be investigated by using specific approaches. At the beginning, the detection of gene deletions/duplications was mainly based on the use of Southern Blot and FISH techniques. However, both approaches are time consuming, with low throughput analysis, and are not able to detect small intragenic rearrangements. On the other hand, CNV detection is mainly based on the use of array Comparative Genomic Hybridization (CGH), but results provided by this approach must in some cases be validated by other quantitative PCR methods, such as microsatellite genotyping, long-range PCR or different array CGH or genotyping platform [4].

Among the different approaches used in recent years for the detection of gene deletions/duplications or for the validation of array CGH results in the analysis of CNVs, particular interest has been devoted to the Multiplex Ligation-dependent Probe Amplification (MLPA) assay (Table 1) [6]. This technique is able to analyze in a single reaction up to 50 DNA sequences and to detect copy number variation of specific genes, including small intragenic rearrangements. So far, over 300 probe sets are commercially available from MRC Holland [6], specific for a very large range of common and rare genetic disorders. MLPA assay has become in a few years a widely used technique in laboratories performing genetic testing for the molecular diagnosis of several diseases. A search in the Pubmed database using the word “MLPA” displays the presence of a total of 978 scientific articles, of which 45 in 2005, 74 in 2006, 124 in 2007, 170 in 2008, 163 in 2009, 229 in 2010, and 203 up to October 2011, thus demonstrating the growing interest devoted by the scientific community to this technique. In this review, we will describe the principles of the MLPA technique and the main applications of this assay in the molecular diagnosis of the most important congenital and acquired genetic diseases.

## 2. Principles of MLPA Assay

MLPA is a multiplex PCR assay that utilizes up to 40 probes, each specific for a different DNA sequence (mainly exons of a specific gene of interest), to evaluate the relative copy number of each DNA sequence. Each probe is composed of two half-probes (5′ and 3′ half-probes), consisting of a target-specific sequence and a universal primer sequence allowing the simultaneous multiplex PCR amplification of all probes [6]. In addition, one or both half-probes contain a stuffer sequence allowing differentiation during electrophoresis of the length of the probe itself, and, as a consequence, the size of the amplification product. The MLPA reaction can be divided into five steps: (1) DNA denaturation and probes hybridization; (2) ligation reaction; (3) PCR amplification; (4) separation of amplification products by electrophoresis; (5) data analysis. In the first step, the DNA is denatured and incubated with a mixture of MLPA probes. The two half probes are able to recognize contiguous target-specific sequences, and only in the presence of a perfect match without a single gap, after hybridization, can the two half-probes be ligated and amplified. PCR amplification is performed using only one PCR primers pair, one of which is fluorescently labelled. Because only ligated probes will be amplified during the subsequent PCR reaction, the number of probe ligation products is a measure of the number of target sequences in the sample. PCR products are then separated by size using Capillary Electrophoresis under denaturing conditions. The height or area of the PCR derived fluorescence peaks is measured, quantifying the amount of PCR product after normalization and comparing it with control DNA samples, thus indicating the relative amount of target DNA sequence in the input DNA sample [6,7]. The quality of the reaction is assessed by the presence of control peaks providing information about the efficiency of the amplification and the correct amount of DNA used for the reaction. A key point in the MLPA reaction is that PCR does not amplify the target sequences, but the ligated probes. Thus, a single pair of PCR primers is used for the amplification, while typical multiplex PCR requires the use of specific PCR primers for each target sequence.

A crucial point in the use of MLPA assay as a genetic test for the molecular diagnosis of gene deletions/duplications is the interpretation of the MLPA results. Homozygous or hemizygous deletions are clearly evidenced by the absence of the specific peaks for the target gene, in the presence of a normal amplification of control probes. On the other hand, heterozygous deletions, duplications and CNVs produce a different height and/or area of the relative peaks, and the interpretation of these results can be challenged by the presence of different efficiencies of the PCR reaction among the different probes and sample-to-sample variations. As a consequence, different MLPA data analysis strategies have been developed to allow a correct interpretation of the reaction raw data. Among these, the most widely used is the Coffalyser software, an Excel-based program able to perform all data normalization steps and corrections for signal sloping Also other software have been recently released [8–10].

## 3. MLPA Applications in Genetic Testing

### 3.1. MLPA and Neuromuscular Disorders

Several types of inherited neuromuscular disorder are due to deletions or duplications of specific genes. Among these, Dystrophinopaties (Duchenne Muscular Dystrophy, DMD, and Becker Muscular Dystrophy, BMD), Spinal Muscular Atrophy (SMA), Charcot Marie Thoot (CMT) disease and Hereditary Neuropathy with liability to Pressure Palsies (HNPP) represent a large portion of all mendelian neuromuscular disease for which genetic testing is routinely carried out for diagnostic purposes, for the identification of healthy carriers and for the evaluation of the recurrence risk. For this reason, MLPA assay represents a powerful tool for the study of these different conditions.

#### 3.1.1. Dystrophinopathies

**Figure 1:**
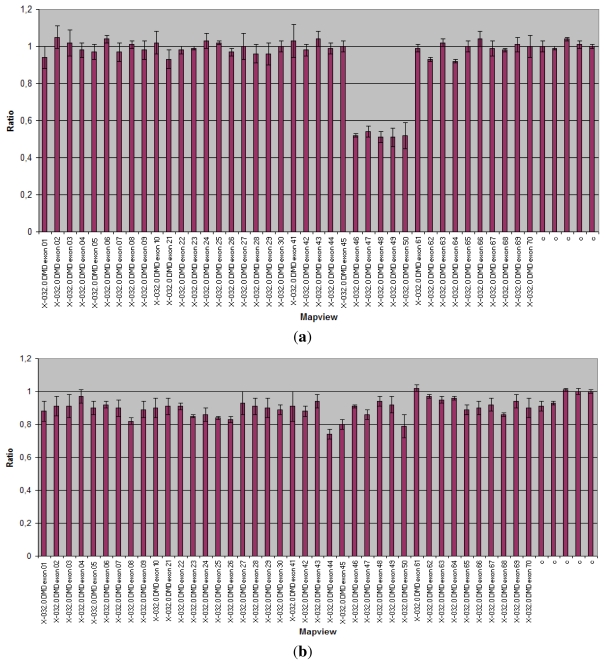
Multiplex Ligation-dependent Probe Amplification (MLPA) analysis of the Duchenne Muscular Dystrophy (*DMD*) gene. Abscissa represents *DMD* gene and control probes (c); ordinate represents fluorescent intensity of amplification. For each probe, the ratio <0.75 stands for deletion; and the ratio >1.3 stands for duplication. (**a**) MLPA analysis showing a heterozygous deletion of exons 46–50 (ratio < 0.75) of the *DMD* gene in the mother of an affected patient; (**b**) Normal control (075 < ratio < 1.3).

DMD and BMD are X-linked diseases affecting 1:3500 and 1:18,000 birth males, respectively, both caused by mutations of the *DMD* gene on Xp21.2. In about 65% of DMD cases and up to 85% of BMD cases the pathogenic mutation is represented by large deletions of the *DMD* gene, while duplications of the same gene account for 5–10% of cases and point mutation are responsible for the remaining 25–30% of cases [2,11–14]. In affected males, about 98% of deletions are easily detectable using a multiplex PCR approach, able to analyze two hot spot regions (exons 2–20 and 44–53) [2,15,16]. However, this approach is not able to detect heterozygous deletions in female carriers, which represents a crucial point for the calculation of the recurrence risk of the disease within a family and the prevention of the birth of affected children. In fact, about one third of DMD cases are due to “*de novo*” mutations in children whose mothers are not healthy carriers and are thus at very low risk of recurrence of the disease. Moreover, *DMD* gene duplications cannot be detected by multiplex PCR approach either in affected males or in female carriers. As a consequence, a number of different approaches has been suggested for the identification of DMD duplications and heterozygous deletions, such as linkage analysis [17,18], quantitative analysis of gene dosage [19,20], FISH analysis [21,22], Entangled Solution Capillary Electrophoresis (ESCE) [23], Primed *In Situ* Labeling (PRINS) combined with FISH [24], Multiplex Amplifiable Probe Hybridisation (MAPH) [25], quantitative real time PCR [26] and CGH array [27,28]. MLPA analysis, based on the use of two SALSA kits able to investigate all the exons of the DMD gene and several control probes on sex chromosomes and autosomes, have been used by several groups in the study of DMD and BMD, both in affected patients and in female carriers [29–35]. All these studies reported MLPA as a simple, rapid and reliable tool in the screening of deletions and duplications of the *DMD* gene, based on its ability to simultaneously hybridize and amplify all of the 79 *DMD* exons in only two reactions tubes, allowing a reduction in labor intensity compared with ESCE, PRINS, real-time PCR and MAPH. The usefulness of MLPA assay is evident in the study of suspected carrier females, where this approach represents a first choice method for the detection of heterozygous deletions/duplications and thus for the assessment of the carrier status in female relatives of affected males (Figure 1).

In the study of affected patients, the MLPA ability to analyze all of the *DMD* exons provides high sensitivity and specificity and a sharp identification of the breakpoints of the rearrangements. This latter represents a crucial point in the management of *DMD* affected patients, since the determination of the full extent of the *DMD* gene deletions/duplications is critical knowledge for possible gene therapy strategies based on the skipping of specific exons involved in the deletion [32]. However, although some authors suggested that the identification of all exons involved in the deletion is critical for predicting the progression of the disease [32], it must be stressed that MLPA analysis is not able to provide information about the “in frame” or “out of frame” status of the deletions, which represents the crucial difference between DMD and BMD causing mutations. The frame-shift mutations in DMD patients result in the complete absence of dystrophin in their skeletal muscle because the translational reading frame of the mRNA is not maintained, whereas muscle tissue from BMD patients contains truncated dystrophin translated from the in-frame mRNA. The difference between “in frame” or “out of frame” deletions can be due to the involvement of even a single nucleotide, and is thus not detectable by MLPA, able to evidence the involved exons but not to identify the specific break points of the deletion.

A crucial point in the interpretation of MLPA results is represented by the detection of deletions involving a single *DMD* exon. In these cases, in fact, the apparent deletion could actually consist of a change in the exon sequence hampering the correct hybridization of the specific probe. This sequence variation can be represented either by a *DMD* pathogenic point mutation or by a polymorphism not affecting gene function. Thus, apparent single exon deletions detected by MLPA should be checked by an independent method [31].

In order to further improve the throughput and speed of the MLPA approach in the diagnosis of *DMD* gene rearrangements, a modification of the original protocol has been described involving the use of a 96-well flow-through microarray system for the detection of the different probes, allowing the hybridization to be completed in 5 to 30 min [36]. In addition, a possible improvement in the detection rate of MLPA analysis is represented by the use of probe multiplexes, including specific probes for common point mutations of the *DMD* gene, allowing both full dosage analysis and partial point mutation analysis in a single test [37].

#### 3.1.2. SMA

SMA (classified in SMA I, II and III according to the severity of symptoms) is a neuromuscular disease characterized by symmetric proximal muscle weakness due to degeneration of the anterior horn cells of the spinal cord. SMA is inherited as an autosomal recessive trait with a prevalence of about 1 in 10,000 newborns and a carrier frequency of 1 in 50 [38]. All the three SMA types are caused by homozygous mutations of the survival motor neuron 1 (*SMN1*) gene (5q13), which in about 95% of cases is represented by the functional absence of this gene due to deletion or its conversion to *SMN2*. This latter gene, mapped within the SMA critical region, is not directly related to the disease, but is considered a disease-modifying gene because its copy number relates to the disease severity and survival of affected patients [39–42].

The standard molecular diagnosis of SMA is based on a PCR-RFLP test, able to detect homozygous *SMN1* loss [43]. However, this method does not detect heterozygous *SMN1* loss, and cannot be used for identifying healthy carriers, which can be checked by linkage or quantitative analysis of *SMN1* copy number. As in the case of the *DMD* gene, also in the case of SMA several additional techniques have been proposed for the identification of healthy carriers, including LightCycler PCR [40], TaqMan Technology [44], and denaturing high-performance liquid chromatography [45].

MLPA assay for the molecular diagnosis of SMA is based on a kit containing several probes for the SMA critical region, including specific probes for *SMN1* and *SMN2* genes, probes able to hybridize both genes and other probes for sequences mapped either within the SMA critical region (NAIP, GTF2H2, *N*-cadherin-like, *CDH6* and *RAD17* genes) or on other autosomal regions. Due to this specific probe set, MLPA assay for the SMA critical region is able to detect the copy number of both *SMN1* and *SMN2* genes. As a consequence, both homozygous and heterozygous *SMN1* deletions or conversions to *SMN2,* can be detected, allowing the diagnosis of affected patients or healthy carriers. Moreover, the assessment of *SMN2* copy number can provide useful information in order to evaluate the genotype-phenotype correlation. Different groups have investigated the efficiency of MLPA in the molecular diagnosis of SMA, both in affected patients and in healthy controls [46–51]. Based on the obtained results, MLPA analysis can be considered as the gold standard technique in the molecular diagnosis of SMA, providing an easy, fast, and high throughput system for analyzing the SMA critical region both in affected patients and in healthy carriers. The advantages of MLPA assay have been particularly stressed in a study of Arkblad *et al*., showing that this technique allowed the identification of a previously unreported, partial deletion of *SMN1* in two apparently unrelated Swedish families, which would not have been detected by conventional diagnostic methods [47]. Due to its ability to simultaneously analyze several samples, MLPA analysis can be used for population screening of SMA healthy carriers in specific conditions, such as in a couple undergoing Assisted Reproduction Techniques. In this light, very recently the usefulness of *SMN1* genotyping in carrier screening for SMA has been suggested by the American College of Medical Genetics, and MLPA approach has been used in this context [52]. The simultaneous analysis of different sequences within and outside the SMA critical region provides an accurate control system, reducing the risk of false positive and false negative results. Moreover, a quick MLPA-based assay for the detection of *SMN1* and *SMN2* copy numbers with high specificity and low complexity has been recently developed [53]. On the other hand, MLPA assay is not able either to detect *SMN1* point mutations or to disclose the presence of two *SMN1* copies in the same allele. However, these conditions account for less than 5% of SMA cases.

#### 3.1.3. CMT and HNPP

**Figure 2:**
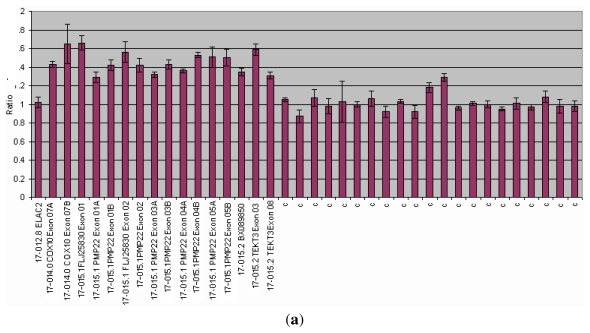

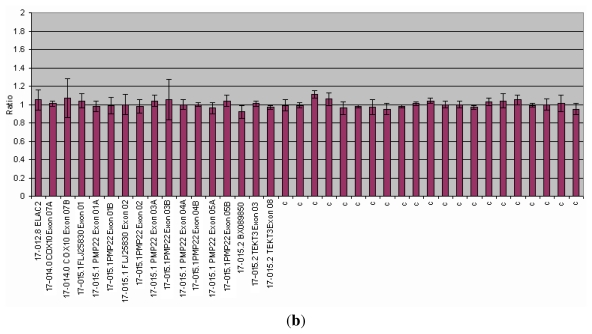
MLPA analysis of the PMP22 gene. Abscissa represents PMP22 gene and control probes (c); ordinate represents fluorescent intensity of amplification. For each probe, the ratio <0.75 stands for deletion; and the ratio >1.3 stands for duplication. (**a**) duplication of the *PMP22* gene (ratio > 1.3) in a patients affected by Charcot Marie Thoot (CMT) disease; (**b**) Normal control (075 < ratio < 1.3).

Charcot-Marie-Tooth disease (CMT) is the most common inherited peripheral neuropathy. Among the different CMT forms, CMT1 is characterized by the presence of demyelinating neuropathies with severe reduction in the motor nerve conduction velocities. CMT1A is the most common type, representing about 70 to 80% of all CMT, and is transmitted as an autosomal dominant trait. The majority of CMT1A cases are caused by a tandem duplication of a 1.5-Mb region encompassing the *PMP22* gene on 17p11.2-p12 [54]. Deletions involving the same gene cause a distinct genetic disease, namely Hereditary Neuropathy with Liability to Pressure Palsies (HNPP) [55]. The incidences of each CMT1A and HNPP are estimated to be as high as 1 in every 2500 individuals [56]. The usefulness of MLPA assay in the detection of *PMP22* duplications and deletions for the molecular diagnosis of CMT1A and HNPP, respectively, has been investigated by Slater *et al.* in a study carried out by comparing the performance of this technique with one of interphase FISH analysis. Authors evidenced a very high concordance of FISH and MLPA, since only one of 50 paired tests produced a false result with FISH analysis, and concluded that MLPA assay represents a robust, simple, and costeffective approach for the molecular diagnosis of CMT1A and HNPP [57]. Thus, this technique is now currently used for the molecular diagnosis of *PMP22* duplications (Figure 2). Moreover, MLPA analysis has been recently used in studies aimed at the identification of alterations of the 17p12 region not involving the *PMP22* gene [58,59].

### 3.2. MLPA and Analysis of the SHOX Gene

The Short Stature Homeobox containing gene (*SHOX*), mapped within the Pseudoautosomal Region 1 (PAR 1) of the X and Y chromosomes, is involved in the regulation of growth and is related to different diseases such as Turner syndrome (TS), Idiopathic Short Stature (ISS), Leri Weill dyschondrosteosis (LWD) and Langer disease (LS) [60]. The majority of mutations causing *SHOX* deficit is represented by deletions within the coding region of this gene or involving a region mapped several hundred kilobases downstream of the coding region and containing conserved non-coding DNA elements (CNE) acting as regulatory elements (enhancer) of *SHOX* [61–64]. *SHOX* mutations affect one to two in 1000 individuals, representing the most common mendelian disease in the Caucasian population [65]. Due to the high frequency of alterations of the SHOX gene and to the recently demonstrated good response to the treatment with growth hormone (GH) in patients with short stature due to *SHOX* deficiency, the early identification of *SHOX* alterations has become crucial not only for the diagnosis of the pathogenesis of the disease, but also for the therapeutic strategy [66–67]. The main approaches originally used for the detection of *SHOX* deletions were FISH or microsatellite analysis, which are both low throughput, time consuming analyses, not useful for application in a screening program, and also considering that short stature is a very common condition affecting about 3% of the population. The usefulness of the MLPA approach in the study of the *SHOX* gene has been tested by different groups, and it has been demonstrated that this approach represents the gold standard for the detection of the *SHOX* gene alterations [64,68–72]. In fact, MLPA is able to detect different *SHOX* gene rearrangements (including small intragenic deletions not detectable by FISH analysis) to evidence the breakpoints of the deletion and to disclose the presence of complex rearrangements involving other genes mapped on the X and Y chromosomes [68]. Moreover, the MLPA probes set for the study of the *SHOX* gene include several probes specific for the *SHOX* enhancer region, being thus able to detect also rearrangements of this regulatory region. Finally, very recently MLPA analyses have also demonstrated that SHOX gene duplications can be responsible for the *SHOX* deficit [72]. MLPA analysis is not able to detect point mutations, but these account for a minor portion of *SHOX* gene alterations and are generally investigated only in second level analysis when the presence of deletions of the coding region or of the enhancer region have been ruled out.

### 3.3. MLPA in Prenatal Diagnosis

Prenatal diagnosis, based on the withdrawal and culture of chorionic villi (CV) or amniotic fluid (AF) samples during pregnancy followed by chromosome investigation, is a largely used assay for the detection of genetic alteration of the fetus. However, two main limits of this approach are represented by the risk of abortion related to the villocentesis or amniocentesis procedures, and the waiting time required for the culture and analysis of samples. Different methods based on the screening of the mist common aneuploidies on uncultured chorionic villi or amniocytes, such as FISH or QF-PCR, are currently used to provide a first result within 24–48 h, followed up by conventional karyotyping on cultured cells. In recent years, the use of MLPA for the screening of aneuplodies of 13, 18, 21, X and Y chromosomes has been suggested. Slater *et al*. assessed the performance of MLPA analysis for rapid, high throughput prenatal detection of common aneuploidies in a blind, prospective trial conducted on 492 amniotic samples [73]. Authors evidenced no failed tests and the clear identification of all autosomal aneuploid cases. Sex determination was also 100% accurate. Based on these results, authors suggested that MLPA is a rapid, flexible, sensitive, and robust test for prenatal aneuploidy detection. Gerdes *et al*. reported a study on 1593 samples (809 AF and 784 CV) in which prenatal diagnosis was performed by using both conventional cytogenetic investigation and MLPA assay [74]. For the purposes of the study, MLPA analysis was organized for completion and reply within 2 days from receipt of the sample. Authors evidenced no incorrect MLPA results, but 51 out of 1593 MLPA analyses (3.2%) were defined as “inconclusive”. van Opstal *et al*. reported a large prospective study on 4000 AF samples using MLPA in order to detect aneuploidies of 13, 18, 21, X and Y chromosomes, obtaining 3932 conclusive (98.3%) and 68 (1.7%) inconclusive results [75]. Among conclusive results, in 76 cases (1.9%) there resulted a normal MLPA analysis, karyotype investigations disclosed the presence of abnormalities such as structural chromosome aberrations, 69,XXX karyotpye, sex-chromosomal mosaicisms, mosaic aneuploidies different from the investigated ones and mosaicism of an extra marker chromosome. All these kinds of aberrations were not expected to be detected by MLPA analysis. The inconclusive results were due to the presence of blood contamination of the AF sample, an insufficient amount of DNA or to unknown reasons. Guo *et al*. developed a MLPA/rtPCR approach to simultaneously detect trisomies 21, 18 and 13 in a single reaction, and investigated 144 blinded clinical samples including 32 cases of trisomy 21, 11 cases of trisomy 18, one case of trisomy 13, and 100 unaffected control samples, comparing results with karyotype analysis. MLPA/rtPCR correctly detected all cases of trisomy even when present in mosaic, suggesting that this approach may have applicability in noninvasive prenatal diagnosis with maternal blood samples [76]. Very recently, Yan *et al*. developed a method of array-based MLPA containing 116 universal tag-probes covering chromosomes 13, 18, 21, X, and Y, and 8 control autosomal genes to rapidly screen for common aneuploidies. In a blind study of 161 peripheral blood and 12 amniotic fluid samples previously karyotyped, these authors evidenced that 97.7% of samples, including all the amniotic fluid samples, were correctly identified by array-MLPA. Authors evidenced the successful application and strong potential of array-MLPA in clinical diagnosis and prenatal testing for rapid and sensitive chromosomal aneuploidy screening [77]. Thus, MLPA analysis appears to be a good candidate to replace interphase FISH analysis for the screening of the most common chromosomal aneuplodies, although the karyotype investigation still remains the gold standard for a complete prenatal diagnosis.

### 3.4. MLPA and Cancer

Several studies have investigated the usefulness of MLPA analysis in the molecular study of different forms of cancer. The three main applications of MLPA assay in this field are (i) analysis of germ line deletions/duplications in genes related to hereditary cancers; (ii) analysis of somatic deletions/duplications in genes involved in the progression of the disease and to the response to therapy; (iii) analysis of DNA methylation as a mechanism of inactivation of tumor suppressor genes. This last topic will be discussed in a specific paragraph.

#### 3.4.1. MLPA and Hereditary Cancers

Hereditary cancers are those in which the presence of a germline mutation causes a hereditary predisposition to the disease. Among these, the most common types are represented by Breast Cancer (BC) and Ovarian Cancer (OC) due to mutations of the *BRCA1* and *BRCA2* genes, Familial adenomatous polyposis (FAP) due to mutations of the *APC* gene, and Hereditary Nonpolyposis Colorectal Cancer (HNPCC) due to mutations of the genes involved in the mismatch repair. The identification of mutations of the above mentioned genes in patients affected by hereditary cancer and in their relatives is of crucial importance in order to set up specific prophylactic strategies. In the majority of cases, germ line mutations affecting these genes are represented by point mutations; however, in the last year, a number of studies have demonstrated that gene deletions/duplications are detectable in a portion of cases which are negative to the screening of point mutations. Several techniques have been used by different groups for the identification of these rearrangements, including MLPA [78]. Several groups have demonstrated the usefulness of MLPA assay in the analysis of genomic rearrangements of *BRCA1* and *BRCA2*. Hogervorst *et al*. using MLPA evidenced the presence of five distinct *BRCA1* deletions/duplications in a series of 661 families with BC in which the screening of *BRCA1* and *BRCA2* point mutations was negative, suggesting that large genomic rearrangements could account for a large portion (about 27%) of all the *BRCA1* mutations in families with hereditary BC [79]. These data were confirmed by Montagna *et al*., who reported that genomic rearrangements account for more than one-third of the BRCA1 mutations in northern Italian breast/ovarian cancer families as evidenced by MLPA analysis [80]. Subsequently, several other studies corroborated the high frequency of *BRCA1* deletions/duplications in families with hereditary BC/OC, although with variable prevalence [81,82]. Other studies have demonstrated that the detection rate of *BRCA1* and *BRCA2* rearrangements by MLPA increases in selected families, such as in the study reported by Woodward *et al*., who evidenced a high frequency of deletions/duplications in multiple case breast/ovarian families with a young age of onset (*BRCA1*) and in families containing at least one case of male breast cancer (*BRCA2*) [83]. In this view, Veschi *et al*. evidenced a very high carrier detection rate of mutation screening plus MLPA analysis in patients in which a high risk to be a carrier had been assessed by the BRCAPro software [84]. Taken together, all these studies strongly suggest the usefulness of MLPA analysis for the search of deletions/duplications of *BRCA1* and *BRCA2* genes in patients without point mutations of these genes.

The application of MLPA analysis has provided useful results also in the study of large rearrangements of the *APC* gene in patients affected by FAP and their relatives. Bunyan *et al*. detected complete or partial gene deletions of APC in six cases out of 24 patients with FAP (25% of mutation negative FAP; 8% of all FAP) [85]. Michils *et al*., using different techniques, including MLPA, evidenced *APC* deletions in 15% of mutation-negative patients with classical FAP, but not in the attenuated FAP [86]. In other studies, MLPA analysis allowed the detection of rearrangements different from deletions as pathogenic mutations of AFP in FAP, such as duplications or complex rearrangements [87,88]. MLPA assay has been also successfully used for the deletions of large rearrangements of genes of the mismatch repair in HNPCC. Nagakawa *et al*., in a series of 70 individuals at risk for Lynch Syndrome, found 6 deletion cases by MLPA assay which were confirmed and characterized by other techniques [89]. Taylor *et al*. analyzed by MLPA 215 UK patients referred for genetic testing on the basis of a family history consistent with autosomal dominant hereditary HNPCC and found 12 cases with deletions of one or more exons (six involving *MLH1* and six *MSH2)*, providing evidence that the overall mutation detection sensitivity in their series was increased by approximately 50% by the inclusion of MLPA, for an additional testing cost of about 10% [90]. Wang *et al*. investigated 112 patients for large deletions of *MLH1* and *MSH2* by MLPA, detecting deletions in 19 patients (11 in *MSH2* and eight in *MLH1*, respectively) [91]. All these authors concluded that large genomic deletions in both *MSH2* and *MLH1* genes play a considerable role in the pathogenesis of HNPCC and should be part of the routine mutation detection protocols. These data were confirmed by several other reports, and MLPA analysis is now considered as a routine approach in the study of the genetic basis of hereditary HNPCC [85,92–95]. However, also in this case it has been suggested that some apparent deletions of single exons may actually result from single base substitutions or small insertions/deletions in the hybridisation sequence of MLPA probes, and that these alterations should be validated with additional methods [96].

#### 3.4.2. MLPA and Somatic Mutations in Cancer

A wide range of MLPA probe mixes for the molecular characterization of cancer samples are available, mostly aimed at the identification of somatic deletions/duplications in genes involved in the progression of the disease and to the response to therapy. An important advantage in the use of the MLPA assay in this field is provided by the ability of this technique to work on formalin-fixed paraffin-embedded tissue, as demonstrated by van Dijk *et al*. by analyzing DNA isolated from formalin-fixed melanomas previously characterized by CGH. These authors reported that MLPA resulted as a reliable and efficient method to evaluate DNA copy number changes as 86% of the tested loci revealed concordant CGH results, and the discordance mainly involved alterations that were detected by MLPA but not by CGH, likely due to the lower resolution of this latter technique and/or to occasionally false positive MLPA results [97]. Thus, MLPA assay has been largely used in retrospective studies on large series of cancer samples based on the use of paraffin embedded tissues. Due to the large number of reports describing the usefulness of MLPA assay in this field, only a few studies have been selected in this review as examples of different applications. In several studies, MLPA assay has been used for the detection of gene deletions/duplications during the progression of several cancer types, in order to relate the detected aberrations with the progression of the disease. Jeuken *et al*. performed MLPA analysis to detect relevant genetic markers in a spectrum of 88 gliomas, the majority of which were previously characterized by CGH assay. MLPA analysis was able to detect complete and partial loss of 1p and 19q even in samples containing only 50% tumor DNA. Moreover, this assay was able to identify distinct 1p deletions showing different clinically prognostic consequences, in contrast to the commonly used diagnostic strategies such as loss of heterozygosity or FISH. Authors evidenced that the combined use of two MLPA probe mixes allows the identification of markers of high-grade malignancy such as *EGFR*, *PTEN*, and *CDKN2A* in 41 cases analyzed, further increasing the accurate prediction of clinical behavior [98]. Franco Hernandez *et al*. analyzed by MLPA and real-time quantitative PCR gene-dosage of the *EGFR* gene 41 oligodendroglial tumors, evidencing the presence of an overdose (one- to five-fold increase) in 21 samples (52.5% of cases) [99]. MLPA assay has been used also for the study of genomic profiles of ovarian and fallopian tube carcinomas, and it has been suggested that dedicated MLPA sets constitute potentially important tools for differential diagnosis and may provide footholds for tailored therapy for these tumors [100]. Subsequently, the study of genomic profiles by MLPA has been extended to several tumors, such as Multiple Endocrine Neoplasia type I, neuroblastoma, meningiomas, larynx and pharynx carcinomas, melanoma, oligodendrogliomas and glioblastomas, gastric cancers, lung cancer, renal carcinoma and others [101–112].

MLPA assay has been used also for investigations of gene deletion/duplication in leukemias. Buijs *et al*. performed genomic profiling using MLPA in 54 cases with suspected or advanced chronic lymphocytic leukemia (CLL), showing that MLPA is able to detect anomalies when the percentage of mutated cells was greater than 35% [113]. A similar study was carried out by Coll-Mulet *et al*., who performed MLPA in 50 CLL patients to identify multiple genomic CLL-specific targets, comparing the results with those obtained with FISH. Authors evidenced a good correlation between MLPA and FISH results, as most alterations (89%) were detected by both techniques. Only cases with a low percentage (<25%) of cells carrying the alterations were not detected by MLPA, but this technique was able to identify intragenic or small alterations undetected by FISH. Authors concluded that the major advantage of the MLPA assay is the ability to provide a simultaneous analysis of many samples with automated data processing at a low cost [114]. The usefulness of MLPA assay in the study of CLL has been further demonstrated by other studies [115–118], and more recently acute leukemias and mielodysplastic syndromes have also been analyzed, again disclosing excellent accuracy and specificity of MLPA as compared to FISH and providing a clinically robust, high-throughput, highresolution option for detection of abnormalities associated with these diseases [119,120].

In other cases, MLPA assay has been used for the detection of gene deletions/duplications related to the response to therapy of specific cancers. In this respect, the MLPA assay has been used for the determination of the status of the *HER-2/neu* transmembrane tyrosine kinase receptor, which represents a prognostic marker and a therapeutic target for breast cancer, being considered a prerequisite for selecting breast tumors for immunotherapy or for taxan based chemotherapy [121]. MLPA results obtained on a group of 60 breast cancer patients were compared with those provided by immunohistochemistry (IHC), showing a good correlation between *HER-2/neu* gene amplification detected by MLPA and overexpression by IHC in invasive breast cancer. Authors concluded that MLPA is an attractive method for detecting *HER-2/neu* amplification in daily laboratory practice [121]. A similar study was carried out by Moerland *et al*., who evaluated the detection of *HER2* gene amplification using MLPA in comparison with FISH on a series of 46 formalin fixed paraffin embedded breast carcinomas, previously tested for protein overexpression by HercepTest. All but one FISH positive cases (18/19) were confirmed by MLPA for the presence of the gene amplification, with a 98% overall concordance of detection of *Her2* gene amplification by FISH and MLPA. Also in this case, authors concluded that MLPA is a reliable and reproducible technique that can be used either as an alternative or additional test to determine *HER2* status in breast carcinomas [122]. These results were confirmed on a larger series by the same group, who compared MLPA, FISH and chromogenic *in situ* hybridization (CISH) in the assessment of the *HER-2/neu* gene amplification status with protein overexpression by IHC in 518 breast carcinoma patients. Authors evidenced that about 10% of their cases overexpressed *HER-2/neu* at the protein level (IHC), and 11% of cases showed geneamplification by MLPA. A high concordance was found between FISH and CISH, MLPA and IHC, and MLPA and CISH, confirming that MLPA is a fast, accurate and cheap method to detect breast cancer *HER-2/neu* amplification in small quantities of DNA extracted from paraffin blocks, and thereby a reliable alternative to FISH and CISH [123]. The same authors also demonstrated an increase in the concordance between MLPA and ISH from 61% to 84% after manual microdissection of the tumor sample and to 90% after laser microdissection, suggesting that microdissection before MLPA may be advisable in the case of very low tumor content or when MLPA results are equivocal [124]. More recently, Moelans *et al*. evaluated the usefulness of MLPA assay for the simultaneous testing of *HER-2/neu* and *TopoII* alpha gene amplification status in 353 paraffin-embedded breast cancer samples, since *TopoII* amplification status determines the anthracycline sensitivity, showing *TopoII* alpha amplification in 9% and *HER2* in 13% of patients, respectively. The authors concluded that MLPA is an easy and accurate method to simultaneously detect breast cancer *HER-2/neu* and *TopoII* alpha copy number status in paraffin-embedded tissue, and thus an attractive supplement or alternative to CISH [125]. Taken together, all these reports clearly demonstrate that MLPA assay can be considered a powerful tool in the molecular analysis of different neoplasm.

### 3.5. MLPA and DNA Methylation

A different application of MLPA analysis is one aimed at the identification of epigenetic alterations, that is modification in the pattern of DNA methylation of specific genes. DNA methylation within the CpG islands in the promoter region is associated with transcriptional silencing and is involved in several cellular processes, such as genomic imprinting, X chromosome inactivation, DNA repair, and others. An aberrant DNA methylation of imprinted genes is associated with several inherited human diseases, and somatic “*de novo*” methylation of CpG islands in tumor suppressor genes has been implicated in tumorigenesis [126,127]. The Methylation Specific MLPA (MS-MLPA) assay has been developed in order to detect epigenetic alterations in the genes involved in the above described disorders [128]. In the MS-MLPA assay the sequence targeted by specific probes contains a restriction site for the HhaI endonuclease, able to recognize the unmethylated GCGC sequence. After the hybridization step, the annealed probe mix is treated with HhaI which digests probes hybridized to unmethylated DNA while leaving undigested the probes hybridized to methylated DNA. As a consequence, only these latter probes will be amplified by the PCR reaction. The comparison of the peak size of the methylation specific probes, between a sample and a control, provide information about the levels of methylation of the specific DNA regions targeted by the probes. Due to this ability, MS-MLPA at present represents the gold standard for the molecular diagnosis of several diseases caused by abnormal DNA methylation. One of the most widely used applications of MS-MLPA is related to the molecular diagnosis of Prader Willi syndrome (PWS) and Angelman syndrome, which are the most common genetic diseases due to alterations of genomic imprinting. Both diseases are linked to the chromosomal region 15q11-q13, with involvement of the paternal allele in PWS and the maternal allele in AS, respectively. PWS, affecting 1:15000 live births, is characterized by hypotonia, hypogonadism, mental retardation and feeding difficulties in early infancy, followed by excessive eating in later infancy or early childhood. PWS can be caused by three different mechanism, namely (i) deletion of the paternal allele in the chromosomal region 15q11-q13 (70% of cases); (ii) maternal uniparental disomy (UPD) for the chromosome 15 (25% of cases); and (iii) abnormalities in the imprinting center sequence on 15q11-q13 (5% of cases) [129].

AS is a neurogenic disorder characterized by mental retardation, microcephaly, inappropriate laughter and seizures. AS is caused by a loss of function of the *UBE3A* gene in the same chromosomal region involved in the PWS, 15q11-13. The *UBE3A* deficit is due to deletion of the maternal allele in the chromosomal region 15q11-q13 (70% of cases), the presence of a sequence variant in *UBE3A* (11% of cases), paternal UPD (7% of cases) and variants in imprinting center sequence (3%). A single MS-MLPA probe set is used for the analysis of both diseases, being able to detect all the different alterations of the 15q11-q13 region, which in the past had to be analyzed using different techniques (Southern Blot or FISH for deletion, microsatellite analysis for uniparental disomy, methylation analysis for the alterations in the imprinting center sequence). Several groups have reported their experience with MS-MLPA in the study of PWS, evidencing the usefulness of this technique which resulted in accurate, reliable results, less time consuming compared to other methods, with the only limitation being the inability to differentiate between UPD and imprinting defects [130–133]. MS-MLPA has been successfully used also in the molecular diagnosis of other diseases related to genomic imprint alterations such as Beckwith-Wiedemann syndrome and Silver Russel syndrome [134–137].

The second important field of application of the MS-MLPA assay is the one related to the analysis of methylation-specific inactivation of tumor suppressor genes in cancer. MS-MLPA has been used to analyze promoter hypermethylation of specific genes such as *MGMT, TIMP3* and *CDKN2A* in different tumors [112,138–142]. Moreover, the MS-MLPA assay has been used to simultaneously detect the methylation levels of up to 25 cancer-related genes, evidencing frequent promoter hypermethylation of specific genes in different cancers and demonstrating the association between epigenetic modifications, progression of the disease and response to therapy [103,143–160]. More recently, the MS-MLPA approach has been used to evidence alterations in the methylation status of microRNA-associated CpG Islands in a large series of hereditary and sporadic carcinomas [161]. All these studies strongly demonstrated that MS-MLPA is a powerful tool to analyze epigenetic alterations in cancer for diagnostic as well as therapeutic purposes, also when one considers the ability of this approach to work on archival formalin-fixed, paraffin-embedded samples as well.

Although MRC-Holland offers an ever-growing range of MLPA probemixes, the application of interest may be too rare or not yet sufficiently investigated to warrant the development of a new SALSA MLPA probemix or to add other probes to MLPA probemix. In both cases, it is possible to develop a synthetic probe set for screening a specific region. This represents an efficient, rapid and robust alternative for research (and potentially diagnostic) deletion and duplication screening of multiple genomic loci [162].

### 3.6. Limits of the MLPA Assay

MLPA offers a rapid means of scanning up to 40 loci for gene dosage, and is likely to be widely used in research and diagnostic settings. However, there are still some limitations. Although MLPA is reported to work with only 20 ng and the results not to be related to the amount of used sample DNA, in our experience we have found that 100–200 ng are required for reliable and reproducible results and that MLPA is also sensitive to the kind of sample used for DNA extraction (e.g., blood or buccal swab). Thus, it is recommended to compare different MLPA analyses only by using DNA extracted from the same tissue with the same method. MLPA reactions are more sensitive to contaminants (e.g., PCR inhibitors such as small remnants of phenol) and DNA degradation than conventional PCR.

As described above a crucial point in the interpretation of MLPA results is represented by the detection of deletions involving a single exon. In these cases, in fact, the apparent deletion could actually consist of a change in the exon sequence hampering the correct hybridization of the specific probe. This sequence variation can be represented either by a pathogenic point mutation or by a polymorphism not affecting gene function. Thus, apparent single exon deletions detected by MLPA should be checked by an independent method. However, some probe sets contain probes able to evidence specific point mutations in the gene or for the region of interest. In this case, MLPA is able to discriminate known point mutations, as probes can be designed so that the ligation site is located directly at the site of the point mutation. Ligation will then only occur on non-mutated sequences, resulting in a decreased fluorescent signal in the case of mutated DNA. By adding common point-mutation-specific (PMS)-MLPA probes to dosage MLPA multiplexes, full dosage analysis and limited point mutation analysis can be performed simultaneously without any significant increase in labor [163].

As compared to FISH, MLPA cannot yet be used to investigate single cells. MLPA analysis of DNA samples from cell mixtures will give the average copy number per cell. In the case of tumor analysis, it is difficult to detect deletions of a certain gene if the sample from which the DNA was derived contained less than 50% cancer cells. The presence of mosaicism might go undetected by the test as the presence of normal cells can mask the presence of abnormal cells.

MLPA is also unable to detect balanced rearrangements, since this technique relies on comparing quantities of DNA from a patient against a control, and balanced rearrangements produce a change in the order of DNA sequences, but not in the DNA quantity.

MLPA analysis of some probe sets results in difficult interpretation due to the presence of unreliable probes. This can represent a limit in diagnostic analysis. Moreover, it is necessary to consider since the MLPA signal is affected by probe size and can have inter-individual variations; different data analysis can potentially lead to different results and interpretations. However, this problem can be resolved using in the analysis at least five control samples to compare to pathological ones. It is also recommended that the standard deviation of each probe of the control sample be analyzed to be sure that it is not significant (Tables 1 and 2).

## 4. Conclusions

In only a few years MLPA assay has become one of the most widely used techniques for the molecular investigation of genetic diseases. The large application of this approach is the result of a number of advantages provided by MLPA assay when compared to other techniques. In fact, MLPA analysis is a high throughput analysis, allowing up to 96 samples to be handled simultaneously, with results being available within 24 h. Moreover, MLPA is a multiplex technique, allowing the study of several regions of the human genome in a single reaction. Target sequences are very short (50–70 nucleotides), allowing MLPA to identify single gene aberrations, too small to be detected by FISH. The MLPA reaction can also be carried out on DNA extracted from a buccal swab, providing an easier system of sample collection compared to peripheral blood withdrawal. Finally, compared to array CGH, MLPA is a low cost and technically uncomplicated method. Over 300 probe sets are so far commercially available, dedicated to the study of several human diseases. In this review we have analyzed some of the most common applications of the MLPA assay, but this technique has also demonstrated its usefulness in the study of several other diseases such as Rett Syndrome, α-thalassemia, disorders of sex development, congenital adrenal hyperplasia, idiopathic mental retardation and Parkinson’s disease [164–171]. Due to this wide range of diseases, it is very likely that MLPA analysis will represent in the near future a basic technique for the molecular analysis of genetic disorders, used in all laboratories performing diagnostic genetic testing both as a confirmation tool and as a diagnostic system applicable also to the copy number variation analysis in rare genetic conditions. Moreover in the future MLPA could be applied to large CNV screening, since recent reports have highlighted the possibility that gene copy number variations may play a role in the development of complex disorders and suggested that some of these variations may be very common.

## Figures and Tables

**Table 1:** Comparison between Multiplex Ligation-dependent Probe Amplification (MLPA) Assay and other methods for the detection of gene deletions/duplications.

Method	Advantages	Disadvantages
MLPA	Detects small rearrangements Up to 40 targets High throughput Low cost	Cannot detect copy neutral loss of heterozygosity. May have problems with mosaicism, tumor heterogeneity, or contamination with normal cells.
FISH	Detects balanced rearrangements Detects mosaicism Detects tumor heterogeneity Can quantify multiple copies	Cannot detect copy neutral loss of heterozygosity. Cannot detect small rearrangements (e.g., deletions <100 kb or duplications >500 kb). Limited number of targets and throughput.
Quantitative/Sq-PCR	Detects small rearrangements and even point mutations Can quantify multiple copies Low cost	Test optimization and efficiency is a concern. Limited number of targets. May have problems with mosaicism, tumor heterogeneity, or contamination with normal cells.
Southern blot	Detects small rearrangements Detects mosaicism	Cannot detect copy neutral loss of heterozygosity. Not quantitative. Laborious and time consuming Limited number of targets and throughput.
CGH array	Can detect very small rearrangements Can probe entire genome Low cost per data point	Cannot detect copy neutral loss of heterozygosity. Costly equipment and reagents Low throughput
SNP array	Can detect copy neutral loss or heterozygosity Can probe entire genome Low cost per data point	Cannot detect small rearrangements (e.g., deletions or duplications <100 kb). Costly equipment and reagents Low throughput

**Table 2:** MLPA applications as diagnostic and discovery tool.

Disease	Gene	Application	MLPA Advantages	Proportion of Cases Due to del/dupl	MLPA Detection Rate of del/dupl	References
DMD/BMD	*DMD* gene	Diagnosis	All the 79 exons of the DMD-gene analyzed in two reactions. Detection of duplications and heterozygous deletions.	60 70% del 5 10% dupl	>99%	[25,29,37]
SMA	*SMN1-SMN2*	Diagnosis	Detection of heterozygous SMN1 loss. Ability to discriminate between SMN1 deletions and conversions to SMN2.	95%	>98%	[46,51]
CMT/HNPP	*PMP22*	Diagnosis	All the PMP22 exons analyzed in a single reaction. A single probe set able to analyze two different conditions.	Dupl 70% 80% CMT. Deletion 85% of HNPP cases	>95%	[57,59]
LWD	*SHOX*	Diagnosis/Research	Ability to analyze both the SHOX gene coding region and the enhancer region. Detection of partial gene deletions and duplications.	40% del	>80%	[68,72]
Aneuplodies of 13, 18, 21, X and Y chromosomes	-	Diagnosis	A single probe mix for the detection of several aneuploidies.	-	>95%	[73,76]
BC, OC	*BRCA1*, *BRCA2*	Diagnosis/Research	Detection of large gene rearrangements.	15 30%	NA	[79,84]
FAP	*APC*	Diagnosis/Research	Detection of large gene rearrangements.	15 25%	NA	[85,89]
HNPCC	*MLH1*, *MSH2*	Diagnosis/Research	A single probe set for all exons of both genes	MLH1:5% MSH2: 20%	NA	[90,95]
PWS/AS	15q11-q13 region	Diagnosis	A single MS-MLPA probe set used for the analysis of both diseases.	Methylation abnormality 95%	99%	[130,133]

Del = deletions; dupl = duplications; NA = not available; DMD = Duchenne Muscular Dystrophy; BMD = Becker Muscular Dystrophy; SMA **=** Spinal Muscular Atrophy; CMT = Charcot Marie Thoot; HNPP = Hereditary Neuropathy with liability to Pressure Palsies; LWD = Leri Weill Dyschondrosteosis; BC/OC = Breast/Ovarian cancer; FAP = Familial adenomatous polyposis; HNPCC = Hereditary nonpolyposis colorectal cancer; PWS/AS= Prader Willi/Angelman Syndrome
